# Immunomodulating Phytochemicals: An Insight Into Their Potential Use in Cytokine Storm Situations

**DOI:** 10.34172/apb.2024.001

**Published:** 2023-07-19

**Authors:** Abdusalam Abdullah Alarabei, Nur Aimi Liyana Abd Aziz, Nur Izah AB Razak, Razif Abas, Hasnah Bahari, Maizaton Atmadini Abdullah, Mohd Khairi Hussain, Amin Malik Shah Abdul Majid, Rusliza Basir

**Affiliations:** ^1^Department of Human Anatomy, Faculty of Medicine and Health Sciences, Universiti Putra Malaysia, 43400 Serdang, Selangor, Malaysia.; ^2^Department of Pathology, Faculty of Medicine and Health Sciences, Universiti Putra Malaysia, 43400 Serdang, Selangor, Malaysia.; ^3^Department of Biomedical Sciences, Faculty of Medicine and Health Sciences, Universiti Putra Malaysia, 43400 Serdang, Selangor, Malaysia.; ^4^Natureceuticals Sdn Bhd, Kedah Halal Park, Kawasan Perindustrian Sg. Petani, 08000 Sg. Petani, Kedah, Malaysia.

**Keywords:** Cytokines, Cytokine release syndrome, Cytokine storm, Immunomodulation, Inflammatory pathways, Phytochemicals

## Abstract

Phytochemicals are compounds found in plants that possess a variety of bioactive properties, including antioxidant and immunomodulatory properties. Recent studies have highlighted the potential of phytochemicals in targeting specific signalling pathways involved in cytokine storm, a life-threatening clinical condition resulting from excessive immune cell activation and oversupply of proinflammatory cytokines. Several studies have documented the immunomodulatory effects of phytochemicals on immune function, including their ability to regulate essential cellular and molecular interactions of immune system cells. This makes them a promising alternative for cytokine storm management, especially when combined with existing chemotherapies. Furthermore, phytochemicals have been found to target multiple signalling pathways, including the TNF-α/NF-κB, IL-1/NF-κB, IFN-γ/JAK/STAT, and IL-6/JAK-STAT. These pathways play critical roles in the development and progression of cytokine storm, and targeting them with phytochemicals represents a promising strategy for controlling cytokine release and the subsequent inflammation. Studies have also investigated certain families of plant-related constituents and their potential immunomodulatory actions. In vivo and in vitro studies have reported the immunomodulatory effects of phytochemicals, which provide viable alternatives in the management of cytokine storm syndrome. The collective data from previous studies suggest that phytochemicals represent a potentially functional source of cytokine storm treatment and promote further exploration of these compounds as immunomodulatory agents for suppressing specific signalling cascade responses. Overall, the previous research findings support the use of phytochemicals as a complementary approach in managing cytokine storm and improving patient outcomes.

## Introduction

 Medicinal plants have been successfully utilized topically and internally since ancient civilisations to treat various health concerns across cultures.^1^ Specifically, herbal compounds have been crucial in drug development, particularly in the treatment of cancer and infectious disorders.^2^ Phytochemicals refer to substances or chemicals derived from plants with unique structures and activities.^3^ These substances are vital for plant development, physiological activities, and defence.^4^ Phytochemicals are abundant in vegetables, fruits, nuts, and seeds.^5^ Researching into the phytochemical family is an enormous undertaking due to its diversity.

 Reports have documented the antioxidant properties of phytochemicals.^6^ Some phytochemicals were reported to precisely modified signal transduction processes, including regulating antioxidant enzyme synthesis and promoting antioxidant effects in cells.^7^ In addition, the antioxidative properties of phytochemicals are essential in preventing neurological disorders, such as Alzheimer’s disease, by minimizing oxygen radicals, neutralizing carcinogenic metabolism, treating and impeding oxidative stress-induced chronic illnesses.^8,9^ Studies have also found that some phytochemicals prevented carcinogenesis, combat microbial infections, inhibited ATP synthase, and promoted skin regeneration.^10,11^ Advancements in plant extraction technology have turned phytochemicals into a more effective, safer and potentially vital components in the development of plant-based medicines.^12^ It is worth noting that approximately one-third of the drugs currently approved by the Food and Drug Administration (FDA) were derived from plants, underscoring the extensive utilization and advantages of medicinal plants.^13^

 Phytochemicals offer several benefits in modulating immune functions, including the maintenance of health through immune system support and the modulation of essential cellular and molecular interactions within the immune system. Cytokines are proteins produced by immune cells and play diverse biological roles. They play crucial roles in coordinating innate immune response by promoting local protective inflammation in acute phase responses.^14^ Cytokines are instrumental in initiating and regulating adaptive immune responses.^15^ Consequently, this review explores the valuable proof and evidence of phytochemicals in regulating immune responses, particularly in the context of cytokine storms.

 Lymphocytes and macrophages are the primary sources of pro-inflammatory cytokines.^16^ In certain disease conditions, an excessive and uncontrolled systemic hyperinflammatory response can occur, which is characterized by elevated levels of pro-inflammatory cytokines such as interleukin-1 (IL-1), IL-6, interferons (INFs), and tumor necrosis factor-alpha (TNF-α). This phenomenon is known as a cytokine storm or cytokine release syndrome, which can lead to multiple organ dysfunction and development of acute respiratory distress syndrome (ARDS).^17-20^ Cytokine activation is nevertheless beneficial in combating infections and malignancies but could be detrimental to the host if released excessively.

 Cytokine storm is a rapidly developing and life-threatening clinical condition. Historically, the term cytokine storm was invented by Ferrara et al^21^ in 1993 to describe the clinical manifestation of graft-versus-host disease. The phrase was later employed in numerous inflammatory diseases, including autoimmune conditions, organ transplantation, as well as in the context of cancer chimeric antigen receptor (CAR-T) cell therapy, which was related to the symptoms that followed the treatment of certain blood cancers,^22^ and. Currently, the term “cytokine storm” has gained increased attention, particularly in infectious diseases such as influenza, severe acute respiratory syndrome coronavirus (SARS-CoV), and coronavirus disease 2019 (COVID-19).^22-28^ This is due the implication of cytokine storm on the severity of these diseases.

 The oversupply of inflammatory cytokines and unrestrained immune cell activation during cytokine storm could result in various pathological conditions, such as continuous fever, arthralgia, myalgia, capillary leak disorder, hypotension, hemophagocytic lymphohistiocytosis (HLH), ARDS, and multi-organ failure.^29^

 Clinical data from several studies on COVID-19 infection have proven that cytokine storm is life-threatening if untreated. The phenomenon is also one of the leading fatal causes of COVID-19.^30-32^ Numerous types of cytokines have been reported to contribute towards the pathogenesis of cytokine storms, thus a single drug treatment might be ineffective. Pathophysiological characteristics of cytokine storm could arise from the effects of pro-inflammatory cytokines, including TNF-α, IL-1, IL-4, IL-6, IL-7, IL-18, and IFN-γ.^33^ Accordingly, effective cytokine storm-reducing strategies may require the suppression of hyperinflammatory responses and modulation of the immune responses.^34^

 Relevant keywords were employed in searching multiple data sources which included PubMed, SpringerLink, ScienceDirect, Google Scholar, and Scopus. In this review, the entire content of pertinent articles was acquired to enable the best literature-based resources accessibility. Furthermore, the data were selected based on the significance and strong understanding of the immunomodulating agent extracted from a plant source and their relationship with cytokine release immuno-pathogenicity to provide an unbiased viewpoint.

###  The chemical nature of compounds with immunomodulatory effects

**Figure 1:**
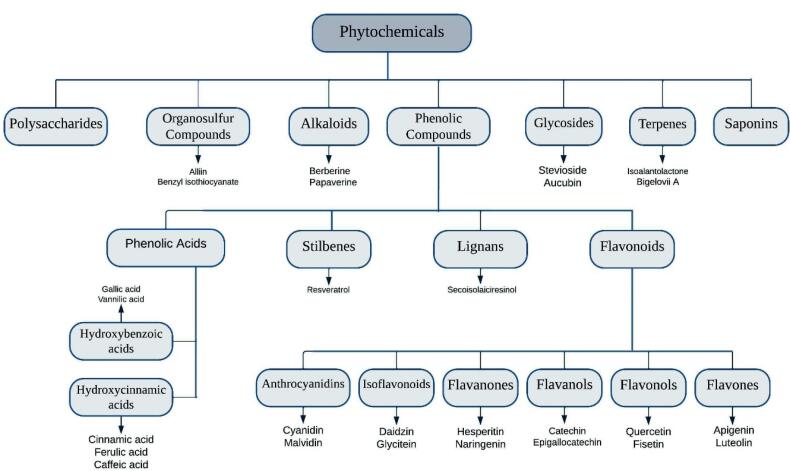
Phytochemicals classification

 Over the years, the interest in natural-derived medicines, particularly phytochemicals, has grown tremendously, resulting in the identification of several active compounds currently categorized as alkaloids, polyphenols, glycosides, organosulfurs, saponin, carotenoids, and terpenes (see Figure 1).^35,36^ Immunomodulators are compounds that regulate or normalise pathophysiological processes.^37^ Researchers have been interested in the immunomodulatory properties of plant- derived phytochemical compounds. Consequently, research investigations on immunomodulatory phytochemicals and their active molecular constituents have led to the development of novel immunomodulatory agents to supplement existing chemotherapies. Nevertheless, most researches have focused on the discovery and investigation of specific families of plant-related chemicals and their potential immunomodulatory effects.^38,39^

###  Polyphenols

 Polyphenols contain at least one aromatic ring with one or more hydroxyl groups, making them one of the largest groups of phytochemicals.^40^ Recent studies reported that phenolic compounds are associated with the positive effects of medicinal herbs.^41^ Phenolic acids, flavonoids, lignans, and stilbenes are the primary subclasses of polyphenols.^42^ In more details, based on the variations in their generic structure, particularly the degree of oxidation of the oxygenated heterocyclic C ring, flavonoids can be classified into different categories, including flavones, flavonols, flavanols, anthocyanidins, flavanones, and isoflavonoids (Figure 1).^43^

 Studies on plant extracts and phytochemicals have demonstrated the anti-inflammatory effects of polyphenols in preventing the progression of chronic illnesses.^44-47^ Polyphenolics also possess superior antioxidant activities, while others such as flavonoids and flavonols demonstrated immunomodulatory actions.^47-49^ Flavanones are another group of polyphenols with an immunomodulatory activity that reduces the intensity of inflammatory responses. Moreover, flavanones extracted from Citrus by-products have been found to possess in vitro antioxidant and anti-inflammatory properties.^50^

 Some polyphenols such as luteolin and quercetin affects the equilibrium between pro- and anti-inflammatory upregulations by suppressing IL-1β and TNF-α synthesis while promoting IL-10 release.^51^ Fouad et al^52^ investigated the immunomodulatory effects of naringenin in acute lung injury (ALI) model in rats. The findings of their study demonstrated that naringenin reduced the expression of TNF-α, nuclear factor kappa B (NF-κB), and inducible nitric oxide synthase (iNOS) and significantly diminished the secretion and action of the pro-inflammatory cytokine (IL-6).

 Zhang and colleagues explored the efficacy of apigenin as a pre-treatment for LPS-induced inflammation in human macrophages. As reported, apigenin markedly inhibited TNF-α, IL-1β-induced NF-κB activation, and IL-6 production.^53^ Furthermore, apigenin has been demonstrated to suppress adhesion molecules [vascular cell adhesion protein 1 (VCAM1) and IL-6-induced intercellular adhesion molecule (ICAM-1)] and chemokines (CCL5).^53^ Consequently, several natural immunomodulating drugs could produce inhibitory efficacy against inflammatory cytokines, thus enabling the possibility of targeting cytokine storm cascades when employed as an immunomodulatory agent during cytokine storm treatments.

###  Alkaloids

 Alkaloids are nitrogen-containing compounds, and are one of the most common phytochemicals in plants. They are found in families such as *Apocynaceae, Amaryllidaceae, Asteraceae*, and *Papaveraceae* families, and have significant biological activities and pharmacological effects due to their nitrogen-containing frameworks with a negative oxidation potential.^54^ Moreover, phytochemicals in this class exhibit anti-inflammatory and antioxidant properties, as well as enzymatic inhibitory activities, which contribute significantly to their role in the treatment of neurological disorders.^55,56^

 Moreover, alkaloids have demonstrated anticancer, antibacterial, and analgesic attributes.^57,58^ Alkaloids, such as colchicine when administered at pharmacological doses exhibited immunomodulatory effects including diminishing cytokines production, such as IFN-γ, IL-1β, IL-6, and IL-18.^59^ Studies also indicated that colchicine suppresses inflammatory cytokines via multiple mechanisms, including the interruption of inflammasome activation [one of the major pathways to limit pro-inflammatory cytokine (IL-1, IL-6, TNF-α) release] and recruitment of more macrophages and neutrophils.^60-62^

 Compared to polyphenols, alkaloids have a narrow therapeutic margin and exhibit activity at extremely low doses. However, their potential cytotoxic effects should not be overlooked, with the exception of chelerythrine and chelidonine.^63^ Additionally, certain alkaloids have been known to cause gastrointestinal side effects, such as diarrhea, nausea, and cramps.^64^

###  Glycosides

 Glycosides are composed of two chemically and functionally independent parts, wherein the glycone (saccharide) portion is linked to another functional group via a glycosidic bond.^65^ Many plants store glycosides as inactive compounds that can be activated by enzymatic reactions.^66^ The pharmacological effects of glycoside are generally attributed to its aglycone moiety, whereas its glycone moiety determines its water solubility.

 Glycosides could be classified based on their aglycone structure, for instance, sterol or flavonoid, whereas the number of saccharides in the carbohydrate unit determines whether the glycosides are mono-, di-, or trisaccharides. Glycosides could also be divided according to the glycosidic linkage between their aglycone and the carbohydrate groups (sugar moiety). Alternatively, therapeutic applications could be employed as another classification basis, such as cardiac glycosides, which are well-known for their positive effects on cardiac arrhythmia.^65,67,68^

 The unique structure of glycosides has resulted in their wide therapeutic applications, including antioxidant, immunomodulatory effects, anticancer, and anticoagulant.^69-71^ Studies have shown that glycosides, such as sativoside, derived from *Stevia rebaudiana* leaves down-regulate pro-inflammatory cytokines production. In addition, it reduces NF-κB and pro-inflammatory cytokines levels IL-1, IL-6, IL-17a, IL-10, and TNF-α.^72-75^ Similarly, naringin, a glycoside derived from the flavanone naringenin and found as the primary bioactive constituent in citrus fruits. This glycoside demonstrated a neuroprotective effect in cerebral infarction by inhibiting neuronal cell apoptosis and diminishing inflammatory cytokines, such as IL-6 and TNF-α.^76^

###  Terpenes (isoprenoids)

 Based on their structure and functions, terpenes are divided into several classes. The isoprene unit is the backbone of terpenes, and most terpenes consist of two or more isoprene units organised in a specific sequence.^77^ Terpenes are basic hydrocarbon structures, while terpenoids are modified terpenes with extra functional groups, commonly relocated or eliminated oxygen-containing groups.^78^

 Terpenes are natural chemical compounds found in plants and animals and are well known for their diverse medicinal qualities. These compounds play a protective role against diseases and parasites in plants, animals, and microbes.^79^ Additionally, terpenes are also essential to plants as they are required in carbon fixation via metabolic reactions.^80^

 Some studies have suggested the potential of terpenes in modulating cytokines due to their lipophilic properties that facilitated their rapid actions and uptakes.^81^ In another study, some terpenes, including carvacrol have shown enhanced production of anti-inflammatory cytokines, such as IL-10.^82^

###  Polysaccharides

 Polysaccharides are macromolecular molecules with broad biological functions. They are composed of more than ten monosaccharides connected by glycosidic linkages. Various polysaccharide molecules have been extracted from organic sources and classified according to their sources as animal, microorganism, or plant polysaccharides.^83^ Recent studies have focused on the immunobiological effects of polysaccharides extracted primarily from Chinese herbal medicine.^84,85^ Several polysaccharide classes have demonstrated antioxidant, antitumor, and immunomodulatory properties. In fact, the most established mechanism is the capacity of polysaccharides to modulate macrophage function. These phytochemicals could inhibit cytokines, including IL-6 and TNF-α, while inducing cytokines, such as IL-2, IL-10, and IL-4. Prospectively, polysaccharides might be the foundation for evaluating new medicinal compounds with immunomodulatory attributes.^86-88^

###  Organosulfur compounds

 Organosulfurs are widely recognized for their exceptional therapeutic characteristics and health benefits. Typically, these class of phytochemicals are found in several dietary sources, including vegetables, fruits, grains, and legumes.^89^ Several plant-based diets rich in organosulfur compounds have been studied for their anti-inflammatory and antioxidant properties.^90^ For instance, *Allium cepa, Allium sativum, *and*Pentadiplandra brazzeana* contain high concentrations of organosulfur compounds such as alliin, allicin, diallyl disulfide, and diallyl trisulfide, which are responsible for the plants’ anti-inflammatory, antioxidant, anticancer, hepato- and cardioprotective properties.^91,92^ Garlic extracts have also been reported to modulate the release of inflammatory cytokines, including IL‐1β, IL-6, and TNF-α, demonstrating immunomodulatory-inducing abilities.^93^

 Studies have revealed that the water fraction of garlic increased IFN-γ and IL-12 levels while suppressing the expression of inflammatory cytokines IL-1, IL-6, and TNF-α in bronchoalveolar lavage fluid.^94^ In another report, allicin inhibited the spontaneous and TNF-α -induced production of IL-1β and IL-8 from two different cell lines in a dose-dependent manner. The diminished cytokine production was attributed to the inhibitory effect of allicin on the breakdown of IκB in the NF-κB pathway.^95^ Therefore, the use of organosulfur compounds alone or combined with other phytoconstituents might be effective against disproportionate immune responses seemingly related to cytokine storm due to their various pleiotropic effects.

Table 1 summarizes the immunomodulatory properties of various natural products with respect to specific regulatory signalling pathways.

**Table 1:** Immunomodulatory properties of various phytochemicals and the associated regulatory signalling pathways

**Phytochemical class**	**Phytochemical name**	**Plant source**	**Experimental model**	**Targeted inflammatory pathway**	**Main effect**	**Reference**
Alkaloid	Berberine	*Coptis chinensis*	LPS-induced ARDS model in mice	NF-κB pathway	Inhibit the production of IL-1β, IL-6 and TNF-α.	^96^
Alkaloid	Berberine	*Coptis chinensis*	Female BALB/c Mice	MAPK and NF-κB signalling pathways	Reduce the expression levels of the relative cytokines IL-2 and IL-4.	^97^
Alkaloid	Protostemonine	*Stemona sessilifolia*	C57BL/6 mice model	MAPK and NF-κB signalling pathways	Decrease generation of IL-1β, IL-6 and TNF-α in murine ALI model. Decrease the expression of iNOS, and the generation of NO.	^98^
Alkaloid	Tetrahydroberberrubine	*Corydalis yanhusuo*	LPS-induced acute lung injury in mice	MAPK, AKT and NF-kB pathways	Inhibit the activation of NF-kB p65 and JNK/p38 MAPKs.	^99^
Alkaloid	Tabersonine	*Catharanthus roseus*	LPS-induced acute lung injury in mice	NF-κB pathway, MAPK/MK2 signalling	Inhibit the production of IL-1β, IL-6 and TNF-α. Inhibit production of iNOS, NO.	^100^
Flavonoid	Cyanidin	Black elderberries, rubus (blackberry, raspberry)	Male C57BL/6 J mice	SirT1/NF-κB pathway	Supress block of NF-B signalling. Reduce IL-1, IL-18 expression.	^101^
Flavonoid	Apigenin	*Cynodon dactylon*, *Mentha longifolia*.	LPS-stimulated human monocytes, LPS-stimulated mouse macrophages.	NF-κB pathways	Suppresses TNF release in primary human monocytes. Reduce the expression of IL-1α, IL-8 TNF-α.	^102^
Flavonoid	Epigallocatechin-3-gallate	*Camellia sinensis* L.	- HPAEpiCs (type II alveolar epithelial cells)/ A549 cells (human alveolar epithelial cell carcinoma), Male ICR mice.	MAPK/STAT3 pathway.	Reduce TNF-α-induced oxidative stress. Suppress MAPKs phosphorylation and expression signal activators of STAT-3.	^103^
Flavonoid	Puerarin	*Radix puerariae*	Male Sprague-Dawley rats	NF-κB/ JAK2/STAT3 Signal	Reduced the levels of IL-1β, IL-6 and tumour TNF-α in cerebral tissue.	^104^
Flavonoid	Luteolin	*Reseda luteola*, other plants	Male Wistar rats	NF-κB pathway	Suppress IL-1β-stimulated inﬂammatory action in rat chondrocytes. Suppress the IL-1β-stimulated phosphorylation of NF-κB p65 in vitro. Decrease the IL-1β-stimulated production of NO, TNF-α, and PGE2. Decrease the expression of iNOS and COX-2.	^105^
Flavonoid	Luteolin	*Reseda luteola*, other plants	Murine model of LPS-induced Acute lung injury.	MAPK/NF-kB pathways	Decrease superoxide dismutase and catalase activity, as well as oxidative damage in lung tissue.	^106^
Flavonoid	Fisetin	Apples, strawberries, cucumbers and many other plants	Male BALB/c mice	NF-kB and NFAT pathways	Inhibit the Th1 and Th2 production, and reduce the ratio of CD8 + / CD4 + T cells.	^107^
Flavonoid	Astilbin	Smilacis Glabrae Rhizoma	LPS-induced ARDS in mice	MAPK signal pathway	Decrease pro-inflammatory cytokines release. Suppressed the activities of myeloperoxidase and malondialdehyde. Supress the expression of TNF-α and IL-6 in vivo and in vitro.	^108^
Flavonoid	Puerarin	*Radix puerariae*	LPS-induced acute lung injury in mice / RAW264.7 cell line	NF-κB pathway	Inhibit the production of IL-1β, IL-6 and TNF-α.	^109^
Flavonoid	Acacetin	*Robinia pseudoacacia* (black locust), *Turnera diffusa* (damiana), *Betula pendula* (silver birch)	sepsis-induced acute lung injury model in mice	NF-κB pathway	Regulate COX-2, iNOS. Decrease pro-inflammatory cytokine concentration.	^110^
Flavonoid	Hesperidin	Citrus fruits	Male BALB/c mice, MCF7 BRCA cell line.	NF-κB pathway	Reduce IL-1 and TNF- levels in the spleen cells. Exhibit good antioxidant& anti-inflammatory properties.	^111,112^
Flavonoid	Hesperetin	Citrus fruits	male C57BL/6J mice	NF-κB pathway	Suppress colitis-stimulated tissue oxidative stress. Suppress TNF-α, IL-6, IL-1β, and IL-33.	^113^
Flavonoid	Hesperetin	Citrus fruits	LPS-induced acute lung injury in mice	MAPK signal pathway	Reduce the number of neutrophils. Reduce the level TNF-α and IL-6, in the model in vivo and in vitro. Regulate IκB degradation.	^114^
Flavonoid	Silymarin	*Silybum* *marianum* (Milk thistle)	human hepatoma cell lines	NF-κB pathway	Inhibit the expression of TNF-α. Exhibits antiviral and anti-inflammatory effects.	^115^
Triterpene	Bigelovii A	*Salicornia bigelovii* T	LPS-induced acute lung injury in mice	NF-κB pathway MAPK pathway	Decrease inflammatory mediators. Neutrophil infiltration.	^116^
Triterpene	Cucurbitacine	*Hemsleya amabilis*	Female BALB/c mice	JAK/ STAT3 pathway NF-κB pathway	Suppress the expression of TNF-α, IFN-γ and IL-6.	^117^
Sesquiterpene	Isoalantolactone	*Inula helenium* L	male C57/BL6 mice	NF-κB pathway	Decrease IL-6, IL-1β, TNF-α, and NO Expression. Suppress neutrophil infiltration.	^118^
Polysaccharides	Kochia scoparia polysaccharide fraction	*Kochia scoparia*	LPS-induced ALI in mice	Not mentioned	Decrease neutrophil infiltration. Decrease IL-6 and TNF-α levels. Reduce neutrophil infiltration.	^119^
Polysaccharides	Dendrobium officinale -extracted polysaccharides	*Dendrobium officinale*	Dextran sodium sulfate -induced acute UC in mice	NLRP3 pathway β-arrestin-1 signal pathway.	Inhibit NLRP3 inflammasome and β-arrestin-1 activation. Reduce the mRNA levels of NLRP3, IL-1β and IL-18.	^120^
Phenolic acid	Gallic acid	Bearberry, pomegranate root bark, and many other plants	C57BL/6J mice	NLRP3 pathway	Decrease IL-1β expression. Inhibit NLRP3 inflammasome activation.	^121^
Phenolic acid	Gallic acid	Bearberry, pomegranate root bark, and many other plants	Male BALB/c mice	NF-κB pathway	Downregulation of TNF-α /IL-1β/ /MIP-2/GCSF genes. Reduce production of IL-1β, IL-6, and TNF-α.	^122^
Phenolic acid	Chlorogenic acid	*Chaenomeles lagenaria*	LPS-induced murine RAW 264.7 macrophages / BALB/c mice	NF-κB/NLRP3 pathway	Reduce production of IL-1b& IL-18.	^123^
Phenolic compound	Imperatorin	*Urena lobata*	Male C57BL/6 mice	JAK/STAT and NF-κB	Decrease the expression of iNOS and COX-2. Inhibit IL-6 and TNFα production.	^124^
Phenolic compound	Isofraxidin	*Sarcandra glabra* and *Acanthopanax senticosus*	Mice in vitro / in vivo	MAPK pathway.	Reduce the production of TNF-α. Regulate proinflammatory cytokines.	^125^
Phenolic compound	Curcumin	*Curcuma longa*	WKY and SHR rats	NF-κB-mediated NLRP3 regulation.	Reduce IL-1β production. Good target for NLRP3 inflammasome-driven disorders.	^126^
Phenolic compound	Apocynin	*Picrorhiza kurroa*, and many other plants.	Adult male SPF Wistar rats	NLRP3 inflammasome Activation. NF-κB signalling NADPH oxidase (NOX) signalling.	Decrease levels ofNLRP3 inflammasome proteins. Reduce the serum level of TNF-α, IL-1β and IL-6.	^127^
Phenolic compound	Paeonol	Moutan Cortex	Trinitrobenzene sulfonic acid TNBS-induced colitis in Female BALB/c mice, colorectal cancer-derived cell line (CW-2)	NF-κB and STAT1	Reduce the production of iNOS protein and mRNA generated by TNF-α and IFNγ signalling. Suppress TNFα-enhanced NF-κB regulation activity and IFNγ stimulation of STAT1.	^128^
Phenolic compound	Gingerol	*Zingiber officinale* (Ginger)	Female Balb/c miceallergy model, HaCaT cell line	NF-κB/MAPK pathways	Suppress inhibited the phosphorylation of MAP kinases. Inhibit the synthesis of cytokines necessary for T cell activation and proliferation.	^129^
Organosulfur compounds	Allicin	Garlic and others	Kupffer cells and male Sprague Dawley rats (treated with acrylamide).	MAPK /NF-κB / NLRP3 inflammasomes pathways	Reduce reactive oxygen species release. Reduced the phosphorylation of JNK, ERK, p65, p38, and IκBα. Suppressing the stimulation of the NLRP3 inflammasome. Reducing the release of IL-1β, IL-6, IL-18, and TNF-α.	^130^
Organosulfur compounds	Benzyl isothiocyanate	*Alliaria petiolata*, and papaya seeds	Male C57BL/6 J mice	NF-κB/NLRP3 pathway	Decrease in IL-1β expression. Reduce macrophage infiltration.	^131^
Organosulfur compound	Alliin	*Allium* species (garlic, onion)	- LPS-induced RAW264.7 cell line, dextran sulfate sodium-induced colitis in ICR mice.	MAPKs-PPARγ /AP-1/ NF-κB /STAT-1 signalling pathways.	Suppress the phosphorylation of p38, JNK. Suppress the transcription of iNOS via interference with STAT-1. Reduce the activity of pro-inflammatory cytokines.	^132^
Glycoside	Bergenin	*Bergenia ligulata* and *Bergenia ciliata*	LPS-induced ALI in male BALB/c mice, Raw264.7 cell line.	NF-κB pathway	Inhibit production of IL-1β, IL-6, and TNF-α. Supress the activation of NF-κB by suppress the phosphorylation of NF-κB p65 unit.	^133^
Glycoside	Stevioside	*Stevia rebaudiana*	Male Wistar rats	-TLR4-MD2 and TNFR1, NF-kB	Reduce the expression of NF-κB and proinflammatory mediators. Free radical scavenger, exert good antioxidant properties.	^74^
Glycoside	Stevioside	*Stevia rebaudiana*	Caco-2 (human colon carcinoma) cell line	NF-κB signalling	Exhibit potent immunomodulatory effects on IκBα activation and NF-κB inhibition and reduce cytokine production. Suppressed LPS-stimulated IL-1β, IL-6, and TNF-α release.	^134^
Glycoside	Catalpol	*Rehmannia glutinosa*	male C57BL/6J mice	JNK and NF-kB signalling pathways	Inhibit JNK and IKKb phosphorylation. Suppress the activation of p50/ NF-kB. Decrease mRNA levels of pro-inflammatory cytokines.	^135^

## Immunomodulating agents and the regulatory signalling pathways

###  Possible pharmacological targets

####  The TNF-α /NF-κBsignalling pathways

 TNF-α is a well-recognized pro-inflammatory cytokine predominantly released by macrophages, monocytes, and T cells. It is implicated in numerous infectious and autoimmune disorders.^136,137^ The TNF-dependent activation of NF-κB also increases anti-apoptotic and pro-inflammatory gene transcriptions.^138^ Furthermore, TNF-α imbalance is the hallmark of numerous autoimmune disorders.^139^ Higher TNF-α concentration has been associated with poor outcomes in SARS-CoV and MERS patients.^140^ Nevertheless, TNF-α has also been reported to inhibit NF-B and ameliorate pulmonary symptoms in mice infected with the SARS-CoV virus.^141^

 The TNF/NF-κB interactions could play pathogenic roles in developing cytokine storm cascades and immune system hyperactivation during cytokine storms. Consequently, suppressing NF-κB signalling pathway could assist in reducing inflammatory diseases.^142^ Selective TNF-α inhibition is also therapeutically helpful in treating different pathological conditions, considering the involvement of numerous other cytokines and intermediates in cytokine storms. Accordingly, TNF-α blockers, such as infliximab and adalimumab, have been used effectively in treating various immune-mediated illnesses. The administration of anti-TNF-α therapy on COVID-19 patients also limits the release of other inflammatory-enhancing mediators. Furthermore, treating patients with active rheumatoid arthritis using anti-TNF-α has resulted in a rapid vascular permeability decrement and reduced broad-spectrum cytokines release, such as IL-6 and IL-1.^143-146^

 Several phytochemicals possess modulatory activation and inflammation ameliorative abilities. For example, quercetin, a polyphenolic component suppresses pro-inflammatory gene expressions by blocking the nuclear translocation of p50 and p65 subunits of the NF-κB receptors.^147^ Min et al^148^ also demonstrated that quercetin diminished the gene expression and production of IL-1β, IL-6, IL-8, and TNF-α in human mast cells by inhibiting IκBα degradation and p65 nuclear translocation. In another report, Chen et al^149^ reported the inhibition of IKK and NF-κB activations, as well as a decrease in NF-κB’s ability to bind DNA in BV-2 microglia mice treated with LPS and IFN-γ.

 Other phytochemicals, such as silymarin (flavonoid),^150^ ursolic acid(triterpenoid),^151^ gingerol (phenolic compounds),^152^ flavopiridol (flavonoid),^153^ zerumbone (sesquiterpene),^154^ curcumin (polyphenol pigment),^155^ and green tea catechins- epigallocatechin-3-gallate (phenolic compounds)^156^ are natural immunomodulatory agents with the ability to block one or more stages in NF-κB signalling. Consequently, pharmacologically profiling of phytochemicals would enable the identification of potent inhibitors for the NF-κB signalling pathway, thus providing a solid rationale for their application in cytokine storm management.

####  The IL-1/NF-κBsignalling pathways

 IL-1β is one of the most investigated IL-1 family members due to its prominent role in autoinflammatory disorders. It is primarily released by macrophages, monocytes, and dendritic cells.^157^ IL-1β derived is from inactive IL-1β precursors via NLRP3 inflammasome cleavage.^158^ Several studies suggested that IL-1β might contribute to the severity of COVID-19 symptoms and autoinflammatory diseases.^159-161^

 In severe COVID-19 cases, reactive oxygen species (ROS) arising from inflammation, and infiltration activates of NLRP3, which is one of the most significant innate immune components. Hence, this process accelerates inflammation by releasing IL-1 and enhancing IL-1 precursor cleavage, which subsequently exacerbates cytokine inflammation throughout the COVID-19 infection.^162,163^ Therefore, a selective antagonist targeting NLRP3 might be a therapeutic target for early-stage disease cases aiming to minimise cytokine storms, alleviate complications, and reduce mortality rates.^164,165^ Moreover, targeting the IL-1RI receptor has been recorded as effective approach during cytokine storm treatments in certain autoimmune disorders, such as CAR-T-cell therapy-induced cytokine storm,^157^ and secondary HLH.^166^

 Numerous phytochemicals have been documented to suppress NLRP3 activation by acting on various stages of inflammasome cascades and positively affecting experimental models. For example, Fan et al^167^ reported that tenuigenin, a triterpene isolated from the root of *P. tenuifolia*, inhibited the activation of NLRP3 inflammasome by repressing ROS before impeding caspase-1 cleavage and IL-1β productions in BV2 microglial cell. Several phytochemicals from different categories have also been found to target NLRP3. Such immunomodulatory agents include triterpenoid Asiatic acid,^168^ sesquiterpene lactone Arglabin,^169^ cucurbitacin,^170^ and iridoid glycoside scropolioside B.^171^

####  The IL-6/JAK-STAT signalling

 IL‐6 is a prototypical cytokine involved in numerous biological processes, including acute-phase reactions, immune responses, and hematopoiesis.^172^ It is characterized by a unique receptor system that consists of two functional proteins: the standard signal transducer for cytokines related to IL-6 (gp130) and the specific receptor for IL-6R.^173^ IL-6 is a good target molecule for cytokine storm given that it is expressed for longer periods than TNF-α and IL-1. It is also considered a superior indicator of disease severity and a prognostic marker for various diseases associated with cytokine storms, including CAR-T-induced and COVID-19.^157,174,175^

 Blockade of IL-6 signalling has produced rapid and significant improvements in clinical symptoms and reduction in serum cytokine levels (including IL-6, IL-8, IL-10, and IFN-γ) during cytokine storms. Therefore, targeting IL-6 antagonism holds promise as a therapeutic approach for various cytokine storms, regardless of the specific situations and cytokine profiles involved.^175,176^

 Gallic acid, a phenolic acid naturally found in vegetables and fruits, has the ability to modulate the activation of the STAT pathway. Pandurangan et al^177^ reported that gallic acid attenuated STAT3 phosphorylation and decreased p65-NF-κB expressions in the colon of mice induced with dextran sodium sulfate. Similarly, Wung et al^178^ revealed the inhibition of IL-6-induced intercellular adhesion molecule (ICAM-1) gene expressions by resveratrol, partly via Rac-mediated pathway interferences through suppression of STAT3 phosphorylation. The safety and efficacy of phytochemicals make them a promising agent to consider for IL-6 JAK/STAT inhibition in cytokine storm therapy.

####  The IFN-γ/JAK/STAT signalling

 IFN-γ signalling plays a crucial role in inflammatory and other immunological responses, contributing to the prevention of viral and bacterial infections.^179^ IFN-γ is predominantly released by NK and activated T cells and is a potent macrophage activator.^180^ Moreover, STAT1 phosphorylation is regulated by JAK1/TYK2 or JAK1/JAK1, which is vital for signalling via the IFN-γ and related receptor class.^181^ Studies have revealed that IFN-γ plays a significant role in several cytokine storm-related diseases.^182^ These findings are supported by evidence indicating that elevated IFN-γ levels are ineffective against infections and lead to immunopathology due to impaired NK function, as evidenced by primary HLH cases.^183^

 Although specific investigations have raised doubts about the role of IFN-γ blockers due to worsened prognosis of severe COVID-19 patients by generating secondary infections in COVID-19 cases, INF-γ blockades could be the core of the treatment.^184^ Numerous studies have reported that several phytochemicals possess potent abilities to reduce or block IFN-γ activation pathways. In a study conducted by Yang and colleagues, it was found that berberine, a natural isoquinoline alkaloid, inhibited the IFN-γ signalling pathway in DSS-induced ulcerative colitis. Regarding mechanisms, berberine regulates the IFN-γ signalling pathway via interaction with the genes responsible for encoding IFN-γ. Furthermore, IRF8 decreased significantly in ulcerative colitis mice treated with berberine.^185^ Ishiguro et al^128^ found that paeonol (polyphenolic product) reduced IFNγ-induced STAT1 activations, TNF-α-induced NF-κB transcriptional activities, and IFN-γ and TNFα-induced iNOS mRNA expressions.

## Conclusion

 Cytokine storm is a life-threatening condition that has been the subject of several studies, aimed at developing immunomodulatory drugs that target specific cytokines. Plant-derived immunosuppressants are a potential alternative for treating cytokine storm syndrome. A combination therapy comprising plant-derived immunosuppressants and some medications may be successful. Therefore, further studies are needed to understand the processes of phytochemically derived immunomodulating agents in different physiological situations and to gain greater insights into their therapeutic applications.

## Acknowledgments

 We gratefully acknowledge the generous support and valuable contribution of NatureCeuticals SDN BHD in funding this research.

## Competing Interests

 The authors declare no conflict of interest.

## Ethical Approval

 Not applicable.

## Funding

 This review article was part of a research project that was supported by NatureCeuticals SDN BHD, which provided financial support for the research.
